# A Review of the Prospective Effects of Methadone on Peripheral Neuropathic Pain in Diabetic Patients

**DOI:** 10.1155/tswj/8483881

**Published:** 2025-03-05

**Authors:** Javad Poursamimi

**Affiliations:** Department of Immunology, School of Medicine, Zabol University of Medical Sciences, Zabol, Iran

**Keywords:** cytokine, diabetes, inflammation, methadone, opioids, peripheral neuropathic pain

## Abstract

Peripheral neuropathic pain (PNP) is a significant complication for diabetic patients, often linked to poor glycemic control and elevated levels of glycosylated hemoglobin (HbA1c). High serum levels of cytokines, such as interleukin (IL)-6, and an increase in T-lymphocytes are crucial factors in developing neuropathic complications. Research suggests that substances like opiates and methadone can provide pain relief for these patients. This literature review is aimed at exploring the advantages and disadvantages of prescribing methadone to individuals with diabetes. We conducted a search of several databases, including PubMed, Google Scholar, Medline, Embase, Web of Science, and Scopus. We used keywords such as “diabetes,” “neuropathic pain,” “methadone,” “opioids,” “inflammation,” and “neuroimmunomodulation.” Ultimately, we identified 19 articles suitable for a more detailed examination. Studies have revealed that the visual analog scale (VAS) index and serum glucose levels decreased in patients who had taken low-dose methadone. Additionally, the production of N-chlorotaurine, a crucial component for innate immunity, was increased in these individuals. Methadone, in a dose-dependent manner, is accountable for increasing serum levels of tumor necrosis factor alpha (TNF-*α*), interleukin-1 beta (IL-1*β*), and interleukin-2 (IL-2) and a high number of monocyte CD14^+^. In conclusion, there were several advantages to taking methadone in a dose-dependent manner, compared to opioids.

## 1. Introduction

Peripheral neuropathic pain (PNP) is a common complication that eventually affects all individuals with diabetes. Reports indicate that more than 2 million Americans suffer from PNP caused by diabetes [1]. Poor glycemic control and elevated glycosylated hemoglobin (HbA1c) levels are closely linked to complications [2, 3], making it essential for patients to manage these factors [4]. Additionally, hypertension, obesity, high blood lipid levels, and smoking are risk factors that can exacerbate symptoms in these patients [4].

As adipose tissue develops, macrophages (MQs) infiltrate it and release proinflammatory cytokines such as tumor necrosis factor alpha (TNF-*α*), interleukin-6 (IL-6), and interleukin-1 beta (IL-1*β*), which have harmful effects on the longevity of beta cells in the Langerhans islets [5–7]. When drug abuse occurs in diabetic patients, T helper 1 (Th1) cells work together with MQs to produce higher levels of TNF-*α*, IL-6, and IL-1*β*. On the other hand, an increase in T helper 2 (Th2) cells and serum levels of anti-inflammatory cytokines (such as interleukin-4 [IL-4]) is likely to reduce diabetic complications [7]. Nowadays, diabetics are considering the use of synthetic or natural drugs such as opioids like methadone and heroin to alleviate pain [5, 7, 8]. This is achieved through the binding of common receptors such as mu (*μ*), delta (*δ*), and kappa (*κ*) with their ligands like methadone [5]. In vivo studies have suggested that these receptors are also expressed in immune cells. However, their interaction with the *μ* receptor on immune cells can have antagonistic effects such as suppressing nuclear factor kappa B (NF-*κ*B) gene expression, interferon (IFN)-*γ* secretion, and antibody production [7, 9]. On the other hand, intracellular synthesis of 3⁣′,5⁣′-cyclic adenosine monophosphate (c-AMP) and secretion of anti-inflammatory cytokines (interleukin-10 [IL-10] and IL-4) are induced [7].

The activity of opioid receptors is dependent on the dose. High doses lead to inhibition of adenylate cyclase and lower intracellular c-AMP levels. This can increase the expression of IFN-*γ* and NF-*κ*B [10]. In this critical review, we aim to clarify these controversial findings.

## 2. Methodology

In this review, 52 articles were extracted from scientific electronic databases including Web of Science, Scopus, Embase, Google Scholar, and PubMed using keywords such as methadone, opiates, diabetes, neuropathy, peripheral neuropathy, immune system, cytokines, innate immunity, cellular immunity, signal transduction, and opioid receptors. Finally, 19 articles were found that can be used for more a precise examination (Table 1).

## 3. Methadone and PNP

The exact mechanism of PNP is unclear. However, the sensation of pain experienced differs from that of cuts or bruises. In neuropathy, damage to the nervous system is not caused by cuts or bruises but rather by a thickening of the capillary membrane in diabetes [27–31].

**Figure 1:**
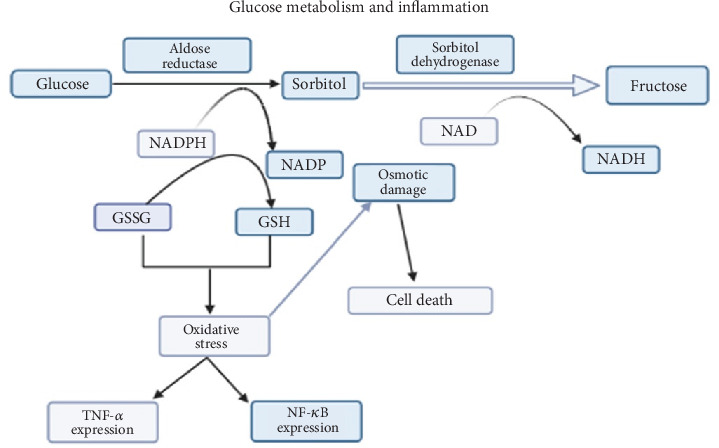
Metabolic pathways of the glucose precursor in DNP.

Microangiopathy or dysfunction of blood capillaries in diabetes may contribute to pain. This occurs when blood glucose levels are not controlled [29]. In such cases, metabolic pathways such as the sorbitol or polyol pathway, oxidative stress, and the production of proinflammatory cytokines are more activated [32] (Figure 1).

Glucose is converted to fructose through the two main enzymes, aldose reductase (AR) and sorbitol dehydrogenase (SDH), of the polyol pathway. When hyperglycemia occurs, the polyol pathway is overactivated, causing an increase in the body's fructose levels and resulting in more free radical production [33].

Some of the free radicals (related to oxidative stress) are synthesized by the enzymatic activity of the reactive oxygen species (ROS) complex in diabetes-induced hyperglycemia [34]. Furthermore, blood levels of free fatty acids (FFAs) and damaged macromolecules such as DNA, lipids, and proteins may also be elevated [34]. Conversely, the antioxidative activity of enzymes such as glutathione peroxidase (GPx) also decreases [35], while serum levels of IL-1*β*, TNF-*α*, and IL-6 increase [36] (Figure 1).

In the following, the sensitivity of adipose and muscular tissues to insulin is compromised. In addition to hyperglycemia, there is also an overactivation of the NADPH oxidase and the protein kinase C (PKC).

**Figure 2:**
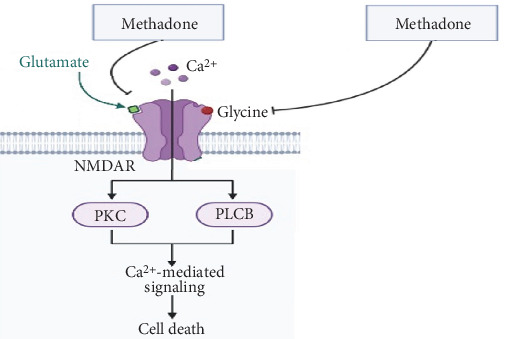
NMDA receptor signaling pathway and methadone.

PKC leads to the activation of p38 mitogen-activated protein kinase (MAPK), extracellular signal–regulated protein kinase (ERK)-1 and extracellular signal–regulated protein kinase (ERK)-2, and NF-*κ*B transcription factor, ultimately increasing the secretion of proinflammatory cytokines [36]. Overall, the pain levels of patients increase. The visual analog scale (VAS) index is typically used for pain assessment. When patients take methadone at a dosage of 2–5 mg daily, it may help alleviate pain and result in a decrease in the VAS score [11, 37]. Methadone occupies the binding sites of N-methyl-D-aspartate (NMDA) receptors related to the amino acids glycine and glutamate, as shown in Figure 2 [11, 38]. Compared to other medications, a low dose of methadone provides a more effective sense of pain control [9, 12, 39] (Figure 2).

## 4. Methadone, Glucose, and Taurine

**Figure 3:**
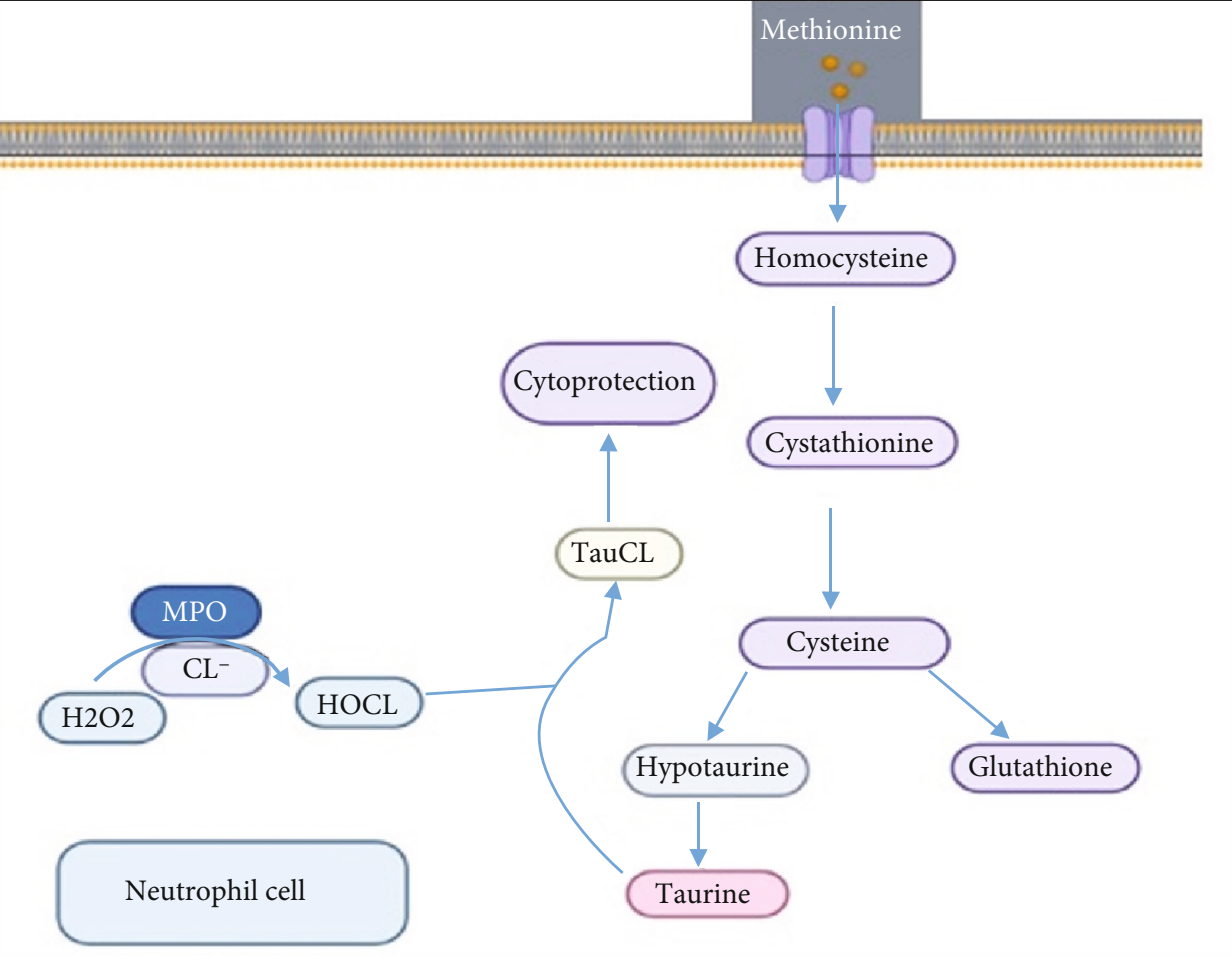
Methionine metabolism pathway and innate immunity.

Metabolism of the amino acid methionine ultimately results in the production of taurine, which plays a role in bile formation, cholesterol excretion, cell volume regulation, ionic transporter activity, inhibition of protein phosphorylation, and innate immunity, even during fetal and neonatal development [40]. Halogen derivatives such as hypochlorous acid (HOCL), produced by white blood cells, may have harmful effects on intracellular organelles. The effects can be neutralized when taurine is converted to N-chlorotaurine [41, 42]. The process of N-chlorotaurine production is illustrated in Figure 3.

If there is a defect in glucose metabolism, such as AR deficiency, sorbitol will accumulate. This accumulation can lead to taurine deficiency, which exacerbates diabetic complications [43]. Studies conducted in vivo have shown that taurine supplements can reduce inflammation in individuals with diabetes [44, 45]. Taurine possesses anti-inflammatory properties by lowering high serum levels of malondialdehyde (MDA), C-reactive protein (CRP), and TNF-*α* and enhancing the activity of antioxidant enzymes such as superoxide dismutase (SOD) and catalase (CAT) [43] (Figure 3).

In addition to diabetes, the sulfur-containing amino acid (taurine) is also secreted into the serum of other conditions including anoxia, myocardial infarction, hepatic encephalopathy, and diseases with osmotic changes. It is interesting to note that serum levels of taurine increase after opioids are taken [17].

Some in vivo studies have shown that methadone and other opioids, which share in the use of *μ*-opioid receptors, have different acts in terms of glycemia. This function was revealed in the use of methadone and tramadol compared with others such as codeine, hydrocodone, oxycodone, hydromorphone, morphine, and fentanyl [46, 47]. Pancreatic insulin release; suppression of counterregulatory mechanisms such as glucagon, epinephrine, and sympathoadrenal responses to hypoglycemia; and impairment of glycogenolysis and gluconeogenesis are some of the likely mechanisms [48]. Furthermore, serum levels of MDA, CRP, and TNF-*α* decreased, while the antioxidant activity of SOD and CAT increased [13, 22, 43].

## 5. Methadone and Osmoregulation

Extracellular and intracellular osmotic perturbations are critical problems faced by all cells leading to cellular swelling and shrinking. Membrane transport processes are essential functions that cells utilize to establish a balance between intra- and extracellular pressure [49]. Ion transport channels such as K^+^ and CL^−^ and Na^+^ and CL^−^ as well as organic osmolytes like polyols (sorbitol and myo-inositol), amino acids and their derivatives (alanine and taurine), and methylamines (betaine) play crucial roles in this process [49]. For instance, when the serum levels of myo-inositol and taurine increase in diabetics, cataracts may develop, as this is related to cellular turgescence in lens cells. Methadone consumption exacerbates the turgescence of lens cells by causing an accumulation of myo-inositol and taurine. This is due to the inactivation of enzymes such as hexokinase, phosphofructokinase-1, and pyruvate kinase, which play essential roles in glucose metabolism [14]. Conversely, heroin inhibits cellular turgescence and reduces neuronal destruction, unlike methadone [17].

## 6. Methadone and Overweight (Metabolic Syndrome)

Although the prevalence of metabolic syndrome has been estimated to be high in heroin or methadone addicts, it also depends on the region, whether it is an urban or rural setting, and the population composition in terms of sex, age, and ethnicity [50]. It is evident that there is a close association between opioid intake, being overweight, and impaired glucose metabolism [19]. The overweight condition in opioid addicts is often due to consuming excessive amounts of sweets, which can also be linked to keeping opioid receptors and channels, such as G protein receptors and ion channels, open. Additionally, methadone can lead to weight gain by activating the *μ* receptors [15, 19].

## 7. Methadone and Sexual Dysfunction

Some sexual dysfunctions, such as low serum levels of testosterone and erectile dysfunction, are commonly observed in subjects taking morphine or methadone [16]. However, some studies have shown that erectile dysfunction in nondiabetics who have taken methadone is not related to serum testosterone levels. This may occur in a dose-dependent manner, so that after reducing methadone consumption, erectile dysfunction improves [18]. Additionally, other factors such as age, hormones, duration of treatment, medical and psychiatric status, and familial conditions can affect erectile function [51].

## 8. Methadone and Immunologic Functions

In a clinical trial involving outpatients with heroin abuse and low immune function, researchers discovered low concentrations of plasma levels of TNF-*α*, IL-1*β*, and interleukin-2 (IL-2) and a low number of monocyte CD14^+^. However, after administering methadone, these factors increased [25]. One of the chemokine receptors found on monocyte cells is CCR5, and it serves as a cofactor in enhancing the pathogenesis of HIV. Studies have shown that individuals who have substance abuse issues or use methadone tend to express high levels of CCR5 on monocytes and dendritic cells. As a result, they often display severe symptoms of HIV infection. This is likely due to the dose-dependent nature of methadone [52]. An in vivo study on the effects of methadone on experimental autoimmune encephalomyelitis (EAE) revealed a reduction in Th1 and T helper 17 (Th17) cell infiltration in the spinal cord, as well as a decrease in levels of IL-2, interleukin-17 (IL-17), IFN-*γ*, and IL-6 in the serum. Conversely, levels of anti-inflammatory cytokines like IL-4 increased [21].

The use of methadone caused a shift in the balance of Th1 to Th2 cells and their respective cytokines. This shift led to a decrease in inflammatory cytokines such as TNF-*α* and matrix metalloproteinase-9 (MMP-9), as well as an increase in the gene expression of anti-inflammatory cytokines and antioxidant enzymes such as CAT and SOD [22].

There are close relationships between hyperglycemia, ischemia, and oxidative stress in diabetic neuropathic pain (DNP). Ischemia occurs as a result of reduced blood flow to the tissue [53]. Reduced peripheral nervous perfusion leads to the release of capillary plasma proteins into the endoneurium, a delicate connective tissue layer surrounding the myelin sheath, causing an increase in capillary wall thickness. This process can lead to hyalinization of the vessel basal lamina and nerve ischemia [54], which can be inhibited using methadone. Therefore, methadone has the potential to reduce the risk of nerve ischemia in diabetes [20]. These conflicting results may be attributed to the dose-dependent manner of methadone.

In general, maintaining homeostasis in diabetes requires the apoptosis process. However, when it is overactivated, it can damage the body. Decreased expression of the BCL-2 gene is a key factor in the apoptosis pathway [55], which may be triggered in monocytes, CD4^+^ T cells, and CD8^+^ T cells using methadone [55].

One of the main components of the immune response is antigen-presenting cells [56], which are weakened in individuals who use opioids [23]. This can lead to low lymphoproliferation and an imbalance in the Th1/Th2 ratio in heroin addicts compared to methadone users [23]. The differentiation, maturation, and activation of dendritic cells are disrupted in opioid addicts [24].

## 9. Methadone and Its Interactions With Other Drugs

The way methadone is processed by the body varies significantly from person to person. Even when given the same dose, different individuals will have different concentrations of methadone in their system. As a result, some patients may not experience enough of the intended effects, while others may have too strong and prolonged of a reaction. Methadone is mainly broken down in the liver to an inactive form. Several cytochrome P450 enzymes are involved in methadone metabolism: CYP3A4, CYP2B6, and CYP2C19. Specifically, certain antiretroviral drugs can lower the levels of methadone in the body. The known drug interactions that affect how methadone is processed usually do not pose life-threatening risks to patients. However, they often lead to lower drug concentrations and effects [57]. The classes of drugs that may be used during methadone maintenance treatment and could potentially interact with methadone include benzodiazepines, antidepressants, anticonvulsants, macrolide antibiotics, and antifungals. Some of the most common ones are listed in Table 2.

## 10. Summary and Conclusions

This study was designed to investigate the effects of methadone on immune responses and signal transduction pathways in diabetic patients. In summary, methadone and other opioids have a variety of effects on the immune system. These effects included the conversion of Th1/Th2 balance to Th2, enhancement of cytokines, reduction of IFN-*γ* and NF-*κ*B expression, and induced intracellular c-AMP synthesis at low opioid and methadone doses. Additionally, there was a decrease in the VAS index, an increase in N-chlorotaurine production in leukocytes, and a decrease in serum glucose levels with low doses of methadone. Other functions of low-dose methadone included amelioration of spinal cord disease and reduction of infiltration of Th1 and Th17 cells into the spinal cord. Methadone and low-dose opioids had anti-inflammatory effects by lowering serum levels of biochemical enzymes such as glutathione (GSH) and mediators (TNF-*α*, MMP-9) and normalizing dendritic cell function.

## Figures and Tables

**Table 1:** Summaries of findings on methadone administration in “in vivo, in vitro, and clinical studies” have been compiled.

**No.**	**First author, year**	**Intervention**	**Results**	**Reference**
1	Amirshahrokhi, 2008	Methadone (10 mg/kg/day subcutaneously) for 24 days	Methadone reduced hyperglycemia and incidence of diabetes, prevented the destruction of *β* cells, and restored pancreatic insulin secretion. It also decreased the Th1 cytokines (interleukin (IL)-1*β*, TNF-*α*, and IFN-*γ*) and increased Th2 cytokines (IL-4 and IL-10)	Animal model [7]

2	Hays, 2002	5 mg methadone every 8 h	Improve daily activities, sleeping, and breathing better. Lower constipation and discontinue gabapentin	Human [8]

3	Bruera, 1995	Methadone was prescribed orally in capsules or suppositories at a dose of 600 mg to cancer patients	In comparison to hydromorphone, methadone was prescribed orally in capsules or suppositories at a dose of 600 mg to patients suffering from cancer pain with a VAS pain index of 10. Patients' pain condition improved. Methadone is an available alternative to hydromorphone as an opioid	Human [9]

4	Gagnon, 2003	Methadone was prescribed 15 mg per day	Methadone at relatively low doses seems to be useful in the treatment of neuropathic pain	Human [11]

5	Dole, 1973	Methadone was prescribed 10 mg per day for 8 weeks	Binding of methadone in body tissues limits the rise in plasma concentration and prolongs the pharmacological action in patients receiving a daily maintenance dose	Human [12]

6	Faskowitz, 2013	A dose of 20 mg/kg methadone subcutaneously induced hypoglycemia in mice	Hypoglycemia was induced in mice using methadone (a dose of 20 mg/kg)	Animal model [13]

7	Sadava, 1997	Methadone was administered orally with a final dose of 1.8 mg/kg for 35 days	Hexokinase and phosphofructokinase 1 activity lowered	Animal model [14]

8	Nolan, 2007	Patients taking methadone were compared with individuals not taking opioids	The mean ± SD of BMI in individuals taking methadone was higher in the control group (those not taking opioids)	Human [15]

9	Cicero, 1975	Three groups of heroin users, methadone users, and a healthy control group were studied. Male secondary sex traits were investigated	It is diagnosed that ejaculate volume, seminal vesicular, sperm motility, prostatic secretions, and serum testosterone levels were reduced by over 50% in the methadone group, as compared to the heroin and healthy control groups	Human [16]

10	Ghandforoush-Sattari, 2010	Thirty heroin addicts were compared to healthy subjects in terms of their taurine blood levels	Plasma taurine level in addicts was more than that in the healthy subjects	Human [17]

11	Brown, 2005	Ninety-two subjects who abused opioids took methadone	Erectile dysfunction, libido dysfunction, and overall dysfunction increased with the patient's age. The dose of methadone showed a significant direct correlation with increased orgasm dysfunction, both before and after adjusting for the duration of treatment	Human [18]

12	Mysels, 2010	Acute and chronic opioid use, alike, concerning public health risks, was investigated. Mu-opioid receptor agonists, such as Methadone, can increase the consumption of sugar-based foods and contribute to a higher BMI in individuals with opioid dependence	Opioid use was associated with weight gain, glycemic dysregulation, and dental pathology	Human [19]

13	Saify, 2015	The human SH-SY5Y cell line was treated with different concentrations (1–2 mM) of methadone and incubated for 24 and 72 h. The cell viability was measured by the MTT dye reduction assay	The expression levels of seven genes (GSTP1, CAT, SOD1, SOD2, NQO1, NQO2, and GSTM2) decreased at 72 h. The mRNA levels of GSTM3 and GSTO2 increased at 72 h There are different pathways for the regulation of antioxidant genes after exposing cells to methadone. Methadone may work through inducing ROS production	Cell line [20]

14	Kafami, 2013	Mice in control and methadone-treated groups received daily intraperitoneal injections of distilled water or methadone (10 mg/kg/day), respectively. Animals were evaluated and scored for clinical signs of the disease from Day 7 to Day 35 after immunization. IL-2, IL-17, IL-6, IFN-*γ*, and IL-10 were measured	Methadone has regulatory effects on the EAE disease process. Methadone diminished the clinical severity of EAE and reduced lymphocyte infiltration into the spinal cord. Methadone may suppress microglial activation and inflammatory cytokine production in the CNS which results in the reduction of demyelination and axonal damage	Animal model [21]

15	Salarian, 2018	Plasma levels of oxidative and inflammatory markers in patients with opioid use disorder and smoking and nonsmoking healthy participants, CAT, GSH, MDA, SOD, MMP-9, and TNF-*α*, were measured	It was observed that lower SOD and catalase activities and higher TNF-*α* and matrix metalloproteinase (MMP)-9 levels in patients were compared to the two comparison groups	Human [22]

16	Sacerdote, 2008	Group A included nine heroin-addicted subjects, who were still injecting heroin; Groups B and C were composed of 12 patients previously addicted to heroin, being treated with methadone (dosage 58 ± 12.7 mg/day) or buprenorphine (dose 9.3 ± 2.3 mg/day) for at least 6 months; Group D comprised healthy controls	PHA-lymphoproliferation was lower in untreated heroin addicts, while it was normal in methadone- and buprenorphine-treated patients. An altered Th1/Th2 balance, characterized by reduced IL-4, IFN-*γ*, and TNF-*α* but normal IL-2 levels, was present in untreated heroin-addicted subjects, while the Th1/Th2 balance was well conserved in the methadone and buprenorphine groups	Human [23]

17	Akbari, 2019	Myeloid DCs (CD11c^+^) and plasmacytoid DCs (CD123^+^) were examined in 20 healthy volunteers and 20 chronic heroin addicts, before and after detoxification with methadone	The percentages of myeloid DCs and plasmacytoid DCs increased in addicted subjects after methadone therapy. The expression of CD11c and CD123 in DC subsets had previously increased, and these changes were further modified after methadone therapy	Human [24]

18	Neri, 2005	Methadone was administered at a dose of 100 mg/day to outpatients with abuse of heroin for 3 weeks	Plasma levels of TNF-*α*, IL-1*β*, and IL-2, as well as the number of monocyte CD14^+^ cells, increased	Human [25]

19	Tahergorabi, 2020	Methadone was administered at a dose of 5 mg/day to rats for 10 consecutive days	Plasma levels of glucose and triglycerides decreased	Animal [26]

**Table 2:** Some of the most common drugs that can interact with methadone metabolism are listed.

**Drug**	**Action**	**Effect on methadone**	**Mechanism**	**Reference**
Amitriptyline	Antidepressants	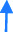 Plasma level	CYP2D6	Vázquez et al. [58]
Carbamazepine	Anticonvulsant	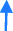 Plasma level	CYP3A4 and CYP2B6	Fuhr et al. [59]
Benzodiazepine	Enhance methadone action such as excessive drowsiness, snoring, and nausea/vomiting	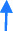 Plasma level	Increase in methadone toxicity (CYP450) (bronchopneumonia and liver disease)	Flanagan and Shepherd [60]
Phenytoin	Anticonvulsants	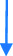 Plasma level	Induction of methadone metabolism (CYP3A4), increase in methadone clearance	Fishman et al. [61]
Fluoxetine	Anticonvulsant	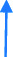 Plasma level	Inhibition of CYP2D6	Fishman et al. [61]

## Data Availability

Data sharing is not applicable to this article as no datasets were generated or analyzed during the current study.
